# The Impact of Perinatal Loss Nursing Simulation among Undergraduate Students

**DOI:** 10.3390/ijerph19148569

**Published:** 2022-07-14

**Authors:** Sook Jung Kang, Yoonjung Kim

**Affiliations:** 1 College of Nursing, Ewha Womans University, Seoul 03760, Korea; sookjungkang@ewha.ac.kr; 2 College of Nursing, Konyang University, Daejeon 35365, Korea

**Keywords:** abortion, miscarriage, nursing student, nursing education, simulation training

## Abstract

Providing careful and proper care for women experiencing perinatal loss is essential. Nurses and nursing students must be sufficiently prepared to provide adequate care. Caring for women who experienced a perinatal loss requires special education and instruction, but little is provided to nursing students. This study aimed to investigate the impact of simulation education directed toward caring for women with perinatal loss. A single-group pretest posttest study design was adopted. A convenience sample of 77 undergraduate students participated in the study. The nursing students’ nursing anxiety, confidence for clinical decision-making, communication competence, and simulative effectiveness were measured before and after the simulation. In addition, we asked students open-ended questions. No significant differences were noted in variables. However, the rank order of simulation effectiveness and result of open-ended questions had some noteworthy implications. Although there was no significant effect in results, simulation education about the perinatal loss was helpful for nursing students. Nursing students reflected on perinatal loss situation and learned that they should provide empathetical therapeutic communication based on needs of women with perinatal loss. To assure its effectiveness and to include optimal program content, a need exists to measure student reflection before and after the simulation. To fully guide and support women and their families with perinatal loss, providing standardized care is needed and in order to do that, standard for educational program regarding perinatal loss needs to be developed and delivered to nursing students as well as nurses.

## 1. Introduction

Some pregnant women experience perinatal loss, the statistics of which vary according to country. However, it is estimated that, every year, approximately 23 million cases of miscarriage occur [1]. This indicates that a perinatal loss including miscarriage, stillbirth, and neonatal loss, which means the loss of a baby, is a common occurrence. Women may experience severe shock, grief, loss, despair, and disappointment before and after a perinatal loss [2], noting that the process is similar to “staying afloat in the storm” [3]. Experiencing a perinatal loss may lead to negative emotion, which is a long-term consequence if the women do not mourn properly [4]. Accordingly, there is a need to provide attentive and proper nursing care to women who experience a perinatal loss. Nurses, especially, are in a good position to support women who suffer from a perinatal loss to prevent negative health outcomes and to help them recuperate physically and mentally from the experience.

Readiness to care for women with a perinatal loss is essential among nurses themselves, because perinatal loss is also a stressful situation for nurses [5] and requires sufficient preparation. In a study of midwives [6], the midwives were found to have low confidence when taking care of women experiencing a perinatal loss. Additionally, in a previous study [6], the instrument of confidence has subscale about knowledge and skills about perinatal loss care, and it found both knowledge and skills were insufficient. Moreover, one of the new nurses’ educational needs include managing unexpected situations such as caring for the ones who have lost their loved ones [7]. In a review by Ellis et al. [8], the attitude and communication skills of healthcare providers were important factors for treating women who have had a perinatal loss. Therefore, relevant education should be provided to nurses and nursing students, including the knowledge and skills necessary to care for perinatal loss.

There is a limited educational resource for nurses who may care for women undergoing a perinatal loss, because it is an interactive process rather than merely providing health information or applying nursing skills. Due to the lack of preparation, nurses experience difficulties with anxiety, powerlessness, managing emotion, and adopting the attitude toward dealing with these patients [9]. Proper nursing, including therapeutic communication, will help the woman with perinatal loss and nurses the process of mourning effortlessly [10]. It may be more efficient if nursing students have the opportunity to learn with a case, especially with a simulated situation.

Simulation training is an effective method for educating students how to treat a clinical scenario [11] without threatening patients’ safety, which takes place in an environment similar to that of a clinical site [12]. According to the standard of best practice, utilizing well-designed simulation is the first step [13]. The topic and content of the simulation scenario need to be closely related to clinical situation as well as congruent to the purpose of the simulation education [13]. Participants can feel the effectiveness and satisfaction of education by going through the process of pre-briefing, simulation operation, and debriefing [13]. When evaluating a well-made simulation, satisfaction is usually measured by the participant’s response, knowledge, and skills acquired through the learning [14]. Simulation education has a prominent advantage that students can repeatedly practice nursing care in a safe environment [15] as well as gain immediate feedback regarding their problem-solving process, which may increase participants’ confidence in real clinical settings [16].

Nevertheless, to date, simulations for perinatal loss are rare. A similar study was conducted to practice neonatal bereavement [17] and bereavement conversation using roleplay [18]. However, previous studies did not identify women who had undergone a perinatal loss. Since it is difficult to conduct practical training related to perinatal loss in real clinical settings, we measured the impact of providing learning opportunities through simulation training for nursing students.

The purpose of this study was to develop perinatal loss nursing care simulation and examine the effectiveness on nursing students’ anxiety and self-confidence for clinical decision-making, communication competence, and simulation effectiveness.

## 2. Methods

### 2.1. Design

A single-group, pretest posttest, quasi-experimental design was used for this study. We conducted a simulation focused on the care of women who have had a perinatal loss as a part of maternal health nursing clinical practice.

### 2.2. Participant

A total of 77 junior nursing students in South Korea participated in this study. The inclusion criterion was nursing students who have completed a maternity nursing class. The sample size required for the research was calculated using G power 3.1. With an effect size of 0.5, significance level of 0.05, and power of 0.8 [19] with on two-tailed Mann-Whitney U test, the minimum sample size for each phase was calculated to be 67. Therefore, this study was explained to all students in the class (82 students) by considering the dropout rate of 20% through the results of previous studies showing that nursing students had a high dropout rate in the simulation study [20]. The study was guided to all 82 students. Five participants were dropped out for personal reasons, and thus, a total of 77 students participated in this study. The collection rate of questionnaires was 94%.

### 2.3. Simulation Operation

#### 2.3.1. Development of Scenario

The research team, composed of a maternal health nursing professor and doctoral student, set up a scenario situation and selected learning goals focusing on nursing care for perinatal loss, an area that does not occur frequently in women’s health, but requires nurses’ preparation. The research team developed the scenario, referring to the healthcare simulation standards of best practice of International Nursing Association for Clinical Simulation and Learning [21]. The scenario was reviewed with a total of 5 experts: professor of maternal health nursing, 3 nurses working in the obstetrics and gynecology ward, and instructor with experience in nursing simulation about the learning goals of the developed scenario and the suitability of the scenario.

#### 2.3.2. Composition and Contents of Simulation

**Figure 1:**
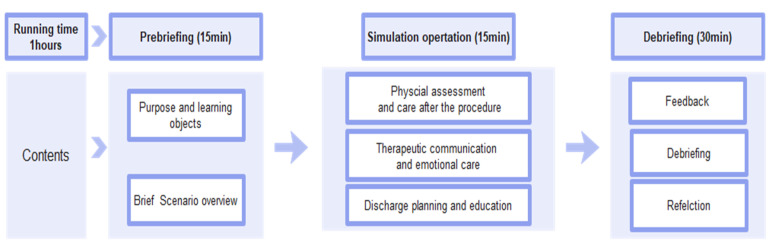
Detailed schedule of simulation.

The simulation was conducted in the order of pre-briefing, simulation operation using high-fidelity manikin, and debriefing. The total running time was about 1 h (Figure 1). In the simulated scenario, women at 15 weeks’ gestation had dilatation and evacuation due to inevitable abortion and had just been transferred to the ward. The scenario consisted of three parts; the first part concerned physical assessment and care after the procedure, including pain control and possible vaginal bleeding. The second part was regarding therapeutic communication and emotional care and dealing with the patient’s worries and guilty conscience. The last part was about discharge planning and education, including answering questions from the patients about future pregnancy. In the simulation operation activity, each team comprised three to four students. One student acted as a caregiver and other students played as nurses. The reason for having two to three nurses in one room was that dealing with this bereavement situation was relatively new to nursing students and interacting with caregiver is an additional task. The instructor acted as a physician through the phone in a control room invisible to students. Before the simulation, the instructors asked students to perform nursing interventions based on the patients’ physical condition and needs. The instructors also asked students to interact with the patients, caregiver (i.e., family member), and attending physician.

### 2.4. Data Collection

Recruitment information materials were distributed in the maternity nursing practicum class. After the researchers explained the simulation to the students in a pre-briefing, only those students who agreed to participate in the study gave written consent and answered the questionnaires. 

In order to protect participant as students, explanations, consent forms, and questionnaires were distributed in envelopes. The explanation of the research was conducted directly by the researcher who did not participate in the lecture, thus ensuring the autonomy of the participants. After the simulation was finished, the researchers were instructed to submit to the box at the back of the classroom while researcher were not present. Participants took about 10 min to fill out the questionnaire. The researcher left the seat while the participants were filling out the questionnaire so that the participants could respond comfortably. It was informed that there is no compensation for participation in the study.

Data were accrued through a self-administered questionnaire at one women’s university in Korea. Before and after the simulation, we measured the nursing anxiety, self-confidence, and communication confidence, and measured the simulation effectiveness after the simulation as well. At the end of the posttest survey, we asked students two open-ended questions: (1) lessons learned from the simulation, and (2) what they would do in real clinical setting.

### 2.5. Instrument

We assessed anxiety and self-confidence levels using the 27-item Nursing Anxiety and Self-Confidence with Clinical Decision Making scale [22]. The instrument comprised two subscales: anxiety and self-confidence. The students provided responses using a 6-point Likert-type scale ranging from 1 (not at all) to 6 (very much so). We determined the quantitative data scores of anxiety and self-confidence levels; high scores represented high anxiety and self-confidence levels in clinical decision making. The Cronbach’s α for the scale was 0.96 and 0.97 for anxiety and self-confidence in the original study [22], and 0.98 and 0.97 for each anxiety and self-confidence in this study. 

We assessed communication competence using Hur’s [23] 15-item modified version of the Global Interpersonal Communication Competence Scale. Students provided responses using a 5-point Likert-type scale ranging from 1 (not at all) to 5 (very much so). Higher scores indicated higher communication competence, and Cronbach’s α for the scale in the original study was 0.89 [23], and 0.74 in this study.

We assessed simulation effectiveness using the 13-item Simulation Effectiveness Tool [24]. Students provided responses using a 3-point Likert-type scale ranging from 0 (Do not agree) to 2 (Strongly agree), and higher scores indicated higher levels of simulation effectiveness. The instrument comprised two subscales: learning and confidence. The total Cronbach’s α for the scale in the original study was 0.88 [24] and 0.91 in this study. The Cronbach’s α for the subscale learning and confidence were 0.87 and 0.88 in the original study [24], and 0.86 and 0.84 in this study.

All instruments used in this study were used with permission from the original author. The instrument for anxiety and self-confidence and simulation effectiveness were translated to Korean language and validity test was accomplished. Translation was carried out by the World Health Organization instrument translation and application guidelines [25]. Then content validity was measured with five experts and Item-level Content Validity Index (I-CVI) was 0.95. and 0.90.

### 2.6. Ethical Consideration

We obtained ethical approval for this study from the institutional review board of the authors’ institution (No.XXX143-13). The research purpose, students’ right to withdraw from the study, and no academic disadvantage were assured if the students refused to participate by the author of this study (i.e., researchers informed the students that the survey would not be linked to evaluations and data will be processed anonymously). Further, participants gave written, informed consent, indicating their willingness to participate. In addition to this, the consent form and questionnaire were distributed in an envelope, and they were allowed to freely submit to the collection box when leaving the class room. When the participants filled out the questionnaire, the researcher was away from the classroom. It was intended to guarantee the voluntary participation and anonymity of the participants. The students were able to stop the survey at any time. The questionnaire will also be stored in a researcher’s office for 3 years after the close of the study and then shredded. There was no compensation for research participation in this study, and the researcher informed the participants in advance.

### 2.7. Data Analysis

Data were analyzed using SPSS 21.0, and statistical tests were conducted with a significance level of 0.05. Descriptive statistics, such as frequency, mean, and standard deviation, were used to assess the participants’ general characteristics and simulation effectiveness. The Mann-Whitney test was used as an alternative to an independent *t*-test, due to the data was not in a normal distribution. The data were abnormally distributed on the Kolmogorov Smirnov test. So, the Mann Whitney U test was conducted for comparing the difference between pre- and posttests. Moreover, qualitative content analysis was performed on the open-ended questions.

## 3. Results

All participants were women aging 20 to 28 years; the mean age of study participants was 21.62 (±1.19) years (Table 1). The majority 54 (70.1%) of students had experience in simulation education before. 13% of the participants (10 students) had prior experience in providing nursing care for women with perinatal loss in the clinical setting. Those who had experience providing nursing care for women with perinatal loss in the clinical setting had more competent communication skills than other nursing students (Z = −2.26, *p* = 0.024).

No significant differences emerged between the pre- and posttest scores for all three outcome measures, such as nursing anxiety and self-confidence for clinical decision-making, and communication competence with the application of the perinatal loss-scenario simulation education (Table 2). The simulation effectiveness score was 17.21 ± 5.57 of 26. The total points and mean of the learning effectiveness subscale were 11.05 ± 3.29 and 1.58 ± 0.47, respectively. Furthermore, the total score and average of the confidence subscale were 4.86 ± 2.05 and 1.21 ± 0.51, respectively. Table 3 shows the rank order of Simulation Effectiveness Tool subscales. The highest score among the items of the learning effectiveness subscale was the item “I was challenged in my thinking and decision-making skills”. The item with the highest score in the self-confidence subscale was “I feel better prepared to care for real patients”.

Participants responded to two open-ended questions (Table 4). The first question was “What have you learned from this simulation education?” for which many students provided the following answers: (a) providing emotional support for women who just experienced a perinatal loss is not an easy task; (b) therapeutic communication is essential for nursing care; and (c) staying calm and careful during nursing care is important. The second question was “How would you care for women who are experiencing perinatal loss in a real clinical setting?” The students mainly answered with the following three points: (a) students would focus on emotional support by having empathy with participants and helping participants express their feelings; (b) students thought emotional support was important, but physical nursing, such as pain control and nursing interventions for hemorrhage, should also accompany emotional support; and (c) students mentioned that there is a need to provide information about the recovery process to women with perinatal loss.

## 4. Discussion

Losing a baby is a traumatic event for women and for their family members. Nurses want to provide optimal bereavement care for the mothers and their families, indicating that education related to this situation is essential [26,27]. However, a paucity of education exists on this topic, and nurses and nursing students do not have enough opportunities to be educated and trained to support and care women in perinatal loss situation [26,27]. Due to this, nurses that work in maternity settings feel that they are not ready for perinatal loss situations, and experience difficulties, such as feeling stress and helplessness, in these situations [28]. One reason for the lack of perinatal loss care might be that the situation is fairly sensitive needing specialized training or knowledge. Hence, in many cases only experienced nurses have the opportunity to care for those women. As a response to that, simulation education is a useful way to enable nursing students and nurses to practice nursing care in a safe environment [12]. In this study, we applied perinatal loss nursing care with simulation education to nursing students and explored its impact.

When evaluating a well-made simulation, satisfaction as the participant’s response and knowledge and skills acquired through learning are measured [14]. Thus, this study measured nursing anxiety, self-confidence for clinical decision-making and communication skills in terms of learning of participants in order to confirm the effect of simulation. Satisfaction was also assessed to measure learner’s responses. Three outcome variables, such as nursing anxiety, self-confidence for clinical decision-making, and communication competence, did not show significant differences after the application of the simulation education. However, according to the open-ended questionnaire, as the participants experienced the situation through the simulation, they realized the difficulty in caring women and their family members with perinatal loss. In some cases, repetitive simulation can increase their nursing competence, such as self-confidence of nursing students [29]. Furthermore, regarding communication competence, sufficient practice is required to use appropriate communication skills in difficult situations such as bereavement [18]. Therefore, experiencing a simulated situation more than one time might be helpful in acquiring the skills and confidence to care for patients with a devastating loss in their life.

We measured the simulation effectiveness after the simulation, and the score was 17.21 ± 5.57, which was similar to the results of a previous study (17.40) that applied maternal health nursing simulation to nursing students in Korea [30]. Some simulation effectiveness items were found to be noteworthy in the rank order. On the learning subscale of simulation effectiveness, an item addressing whether participants were challenged in thinking and decision-making skills scored the highest. This may mean that the simulation on perinatal loss led them to rethink and reflect on their direct nursing care method and clinical knowledge. In future studies, other learning abilities that more closely relate to reflection or critical thinking should be measured. On the confidence subscale of simulation effectives, the item concerning feeling better prepared to care for real patients scored the highest. Helping students better prepare to care for mothers who experience perinatal loss was one of the main purposes of applying this simulation education. Having this item score highest may implicate the positive effectiveness of the simulation education that was applied in this study.

The responses to the open-ended questions imply that participants may need more education or practice on providing emotional care and communicating with compassion with patients. Additionally, students in this study perceived that, in a real clinical setting, both delivering optimal physical care and information about the recovery process is critical. Therapeutic communication is an essential factor in nursing a woman who has experienced a perinatal loss and is in the mourning process. Women who experienced a perinatal loss reflected that they needed physical and emotional care, and had a cold or inhuman experience in the relationship with the healthcare provider [31]. According to the results of the open-ended question, the students thought that they should provide both the physical and emotional care required by the women. In other words, the simulation became an opportunity for the students to perceive that they should be prepared to provide physical and emotional care and therapeutic communication toward caring for women who have experienced a perinatal loss.

As mentioned earlier, caring for a woman with perinatal loss requires not only a delicate and thoughtful approach, but also precise knowledge, so that women experiencing bereavement can recuperate adequately. Structured and interactive nursing simulations about perinatal loss will help prepare students to care for this delicate patient, who has complicated emotions due to the grieving process. Furthermore, a standardized education content that includes specific scenarios for miscarriage, stillbirth, and neonatal loss need to be developed, so that every woman and their family can receive a proper nursing care regardless of medical staffs or medical institutions they encounter. Lastly, having a term and healthy baby does not mean that the previous perinatal loss has been fully recovered. Hence, nurses always need to assess whether there is any remaining emotions or trauma from past experience when caring women and their family who has a perinatal loss experience.

This study has three limitations. First, since the data collection was conducted at a women’s university, the study is limited by how all participants were women, without any male students. Therefore, the study should consider expanding the demographics of its participants in the future. Second, a single-group quasi-experimental design was used for this study. Third, in the case of a participant who had experienced with perinatal loss care, we did not identify how much they have involved in that nursing care a nursing student. Therefore, in the future, we propose a follow-up study with a two-group, quasi-experimental or randomized study as well as identifying the relationship between the involvement and impact of perinatal loss simulation for women and their family.

## 5. Conclusions

The purpose of this study was to examine the impact of simulation training directed toward caring for women who experience perinatal loss on nursing students. Although measured outcomes did not show significant improvements after the application of the simulation, the result of the simulation effectiveness scores and open-ended questions revealed that the simulation had a positive impact on nursing students. Nursing students were able to realize and reflect on perinatal loss through the simulation such as this study. In the process, the students realized that they needed to provide nursing care that fits the needs (i.e., physical, and emotional) of women who have experienced a perinatal loss and conduct empathetical therapeutic communication. Future studies examining the knowledge related to reflection when caring for women with fetal loss need to confirm the efficacy of simulation education of nursing care for women with perinatal loss. Moreover, simulations should be repeated so that the nursing students can become familiar with the situation and practice completely.

## Figures and Tables

**Table 1:** Characteristics of participants (*n* = 77).

Characteristics	Categories	*n* (%)	Mean (SD)
Age (yr)			21.62 (1.19)
Previous simulation experience	Yes	54 (70.1)	
	No	23 (29.9)	
Did you experience a perinatal loss situation during your clinical practice?	Yes	10 (13.0)	
	No	67 (87.0)	

**Table 2:** Comparison of dependent variables between pretest and posttest.

Variables	Pretest		Posttest		Z (*p*)
	Median (IQR)	Range	Median (IQR)	Range	
Anxiety in clinical decision making	90.0 (28.0)	56–153	84.0 (31.0)	41–141	−0.86 (0.399)
Self-confidence levels in clinical decision making	99.0 (22.5)	53–140	104.0 (22.5)	53–153	−1.46 (0.146)
Communication competence	53.0 (6.5)	43–64	53.0 (7.0)	35–63	−0.26 (0.793)

**Table 3:** Rank order of simulation effectiveness tool score (*n* = 77).

Subscale	Item	Mean ± SD	Range
Learning †		11.05 ± 3.29 (1.58 ± 0.47)	1–16 (0.13–2.00)
	I was challenged in my thinking and decision-making skills	1.62 ± 0.51	0–2
	Debriefing and group discussions were valuable	1.55 ± 0.53	0–2
	I learned as much from observing my peers as I did when I was actively involved in caring for the simulated patient	1.53 ± 0.53	0–2
	Completing the simulation clinical experience helped me understand classroom information better	1.40 ± 0.59	0–2
	The instructor’s questions helped me to critically think	1.40 ± 0.52	0–2
	My assessment skills improved	1.31 ± 0.63	0–2
	I developed a better understanding of the pathophysiology of the conditions in simulated clinical experience	1.18 ± 0.70	0–2
	I developed a better understanding of the medications that were in the simulation clinical experience	1.05 ± 0.72	0–2
Confidence †		4.86 ± 2.05 (1.21 ± 0.51)	1–10 (0.20–2.00)
	I feel better prepared to care for real patients.	1.38 ± 0.56	0–2
	I am more confident in determining what to tell the healthcare provider	1.30 ± 0.65	0–2
	I am able to better predict what changes may occur with my real patients	1.18 ± 0.68	0–2
	I feel more confident in my decision making skills	1.18 ± 0.64	0–2
	I feel more confident that I will be able to recognize changes in my real patients’ condition	1.12 ± 0.74	0–2
Total		17.21 ± 5.57 (1.32 ± 0.43)	2–26 (0.15–2.00)

† Subscale of Simulation effectiveness.

**Table 4:** Questions and results of open-ended questions.

What Have You Learned from This Simulation Education?
Providing emotional support for women who just experienced a perinatal loss is not an easy task Therapeutic communication is essential for nursing care Staying calm and careful during nursing care is important.
**How Would You Care for Women Who Are Experiencing Perinatal Loss in a Real Clinical Setting?**
Focus on emotional support by having empathy with participants and helping participants express their feelings Emotional support was important, but physical nursing, such as pain control and nursing interventions for hemorrhage, should also accompany emotional support There is a need to provide information about the recovery process to women with perinatal loss

## Data Availability

The data used to support the findings of this study are included within the article. The data are not publicly available due to privacy or ethical restrictions.
